# Pulmonary Artery Sling Presenting as Pneumonia and Inhalation of a Foreign Body

**DOI:** 10.1155/2019/8912136

**Published:** 2019-02-17

**Authors:** Shujuan Luo, Huaping Rao

**Affiliations:** Hunan Children's Hospital, Changsha, China

## Abstract

Pulmonary artery sling is a rare cause of respiratory distress created by compression of the lower trachea and right mainstem bronchus due to an aberrant origin of the left pulmonary artery. The condition is frequently associated with recurrent respiratory infections and other congenital malformations including tracheal rings. We present the case of an infant presenting with pulmonary distress and a history of recurrent respiratory infection. The infant underwent surgery to remove a foreign object; however, the symptoms did not resolve. Bronchoscopy revealed bronchus stenosis, and subsequent echocardiogram and CT scans revealed the presence of a pulmonary artery sling. We prescribed infection prophylaxis with the immunomodulator OM-85 to mitigate the risk of further infections prior to surgery. PAS and bronchus stenosis were corrected successfully by surgical intervention leading to resolution of symptoms of respiratory distress and a reduction in the incidence of respiratory infection.

## 1. Introduction

Pulmonary artery sling (PAS) is a rare cause of respiratory distress created by an aberrant origin of the left pulmonary artery which results in compression of the lower trachea and right mainstem bronchus. The condition, which is frequently associated with tracheal rings and recurrent respiratory tract infections, is correctable by surgery.

## 2. Case Study

**Figure 1:**
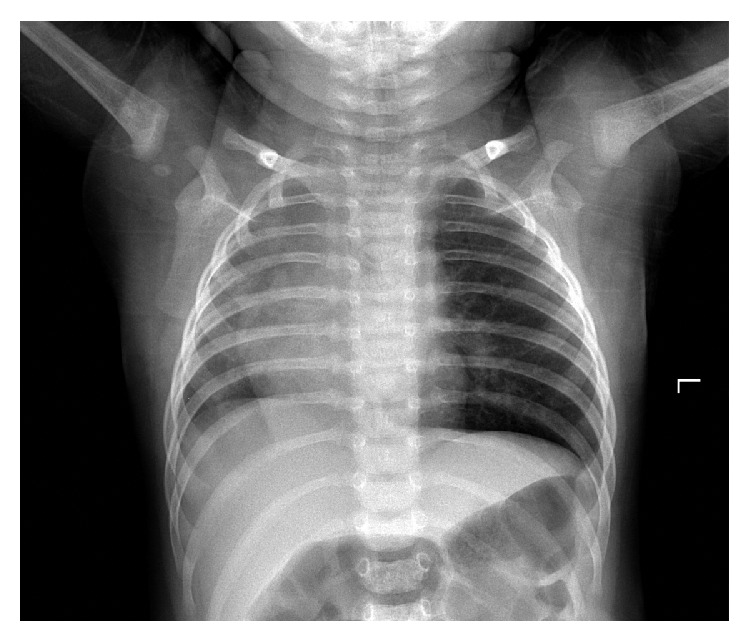
Chest X-ray of a 13-month-old male child showing bronchopneumonia, mediastinum right shift, and emphysema on the left-hand side.

A 13-month-old male child was referred to our ENT outpatient clinic on January 13, 2014, with a 3-day history of gradually worsening cough. The parents stated that the coughing had begun after a fit of choking while the child was eating rice. During initial presentation at a local hospital, auscultation revealed that breath sounds were diminished on the left-hand side. In view of the retching during eating, the attending physician diagnosed the child as having a bronchial foreign body and referred the patient to our clinic. A chest X-ray showed bronchopneumonia, mediastinum right shift, and emphysema on the left-hand side (Figure 1).

**Figure 2:**
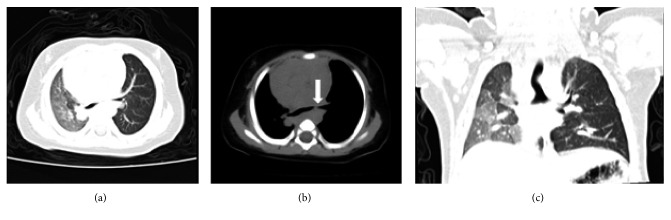
CTVB showing signs of pneumonia and indications of a bronchial foreign body (indicated by arrow).

The attending ENT physician suggested carrying out bronchoscopy using a flex electronic bronchoscope to confirm the foreign body. However, as we were unable to schedule the procedure for 3 days, and in view of high state of anxiety experienced by the parents, the attending physician scheduled computed tomography virtual bronchoscopy (CTVB). The CTVB also showed signs of pneumonia and indications of a bronchial foreign body (Figure 2). Auscultation confirmed reduced breath sounds on the left as well as rales and wheezing.

A decision was made to remove the suspected foreign object under general anesthesia. After preoperative preparation and anesthesia, the surgeon visualized the airways using a rigid electronic bronchoscope. No foreign body was seen in the left bronchus; however, a foreign body resembling rice was visualized in the right bronchus. The rice-like object was removed, and before completing the operation, the doctor confirmed there was no trace of a foreign body left in either bronchi. The postsurgery diagnosis was presence of a bronchial foreign body with bronchopneumonia.

Three days after the operation, the child's cough and wheezing had still not resolved, so the attending physician asked a respiratory specialist for a consultation. The specialist suggested completing a sputum culture test and a viral rapid antigen direct test, with additional medication (i.e., atomization of budesonide, ipratropium bromide, and salbutamol) to treat the wheezing and cough. Two days later, symptoms and signs had improved. However, a routine predischarge chest X-ray revealed no improvement in signs of pneumonia, mediastinum right shift, or emphysema. Unfortunately, at that time, the parents refused further examination and asked for the child to be discharged.

**Figure 3:**
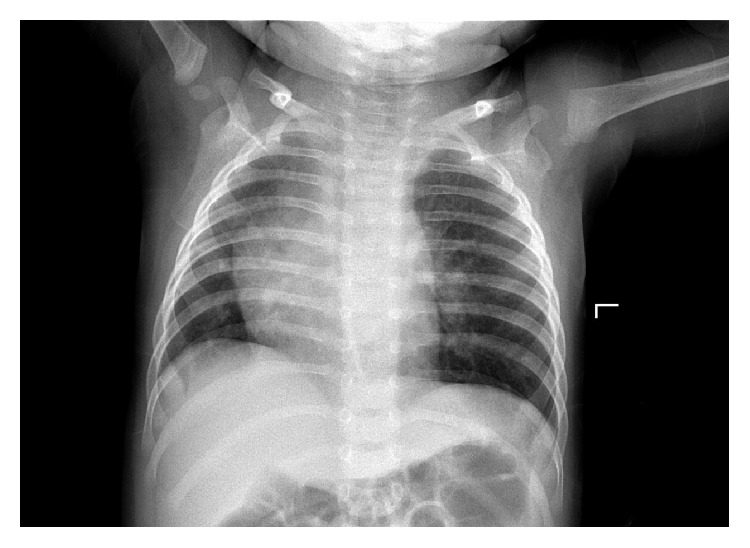
Chest X-ray showing continued presence of bronchopneumonia, mediastinum right shift, and emphysema on the left-hand side.

One month later, the patient was readmitted to our respiratory clinic with a 5-day history of coughing. Cyanosis, fever, and tachypnea were absent. A review of medical history showed the child had experienced 8-9 respiratory tract infections since the age of 3 months (mainly bronchitis or bronchopneumonia). There was no asthma or family history of asthma. A physical examination revealed reduced breath sounds on the left-hand side alongside rales and wheezing. The chest X-ray showed that bronchopneumonia, mediastinum right shift, and emphysema on the left-hand side were still present (Figure 3).

**Figure 4:**
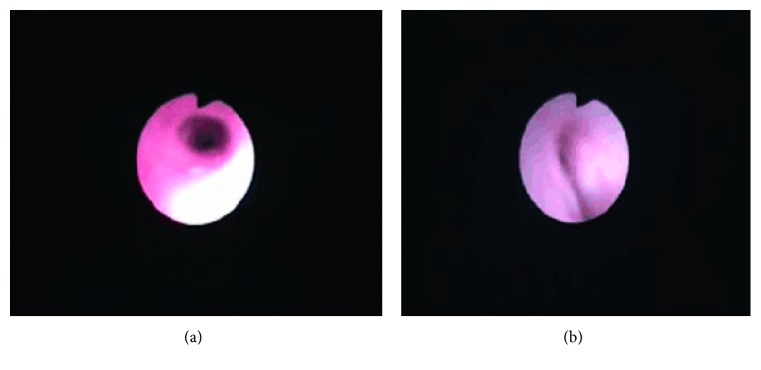
Bronchoscopy showing airway dysplasia: O-type tracheal cartilage (a) and stenosis in the left bronchus (b).

Immunological analysis by turbidimetric inhibition immunoassay showed reduced C3 and C4. A diagnosis of bronchopneumonia and recurrent respiratory tract infection (RRTI) was made. We treated the bronchopneumonia and made an appointment for flex electronic bronchoscopy, which revealed airway dysplasia, O-shaped tracheal cartilage, and stenosis of the left bronchus (Figure 4).

**Figure 5:**
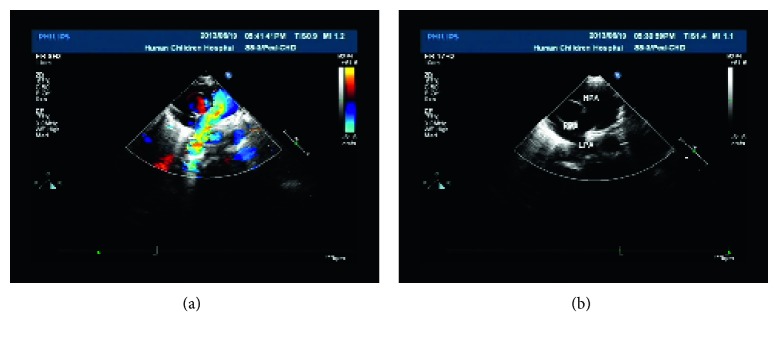
Echocardiograms showing PAS.

**Figure 6:**
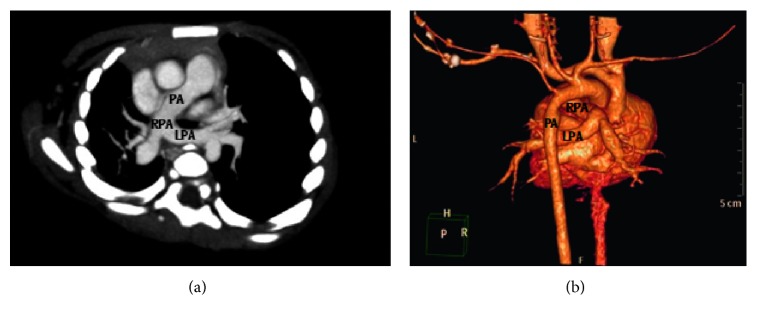
CT angiography (a) and 3D reconstruction (b) showing the anomalous origin of the left pulmonary artery (LPA) from the posterior aspect of the right pulmonary artery (RPA).

In order to differentiate between potential congenital, inflammatory, or vascular compression-related etiologies of the stenosis, an echocardiogram was undertaken revealing a PAS (Figure 5). A further cardiac computed tomography (CT) angiography confirmed the diagnosis (Figure 6). And there was actual left bronchus stenosis, and the stenosis was about 0.8 cm.

Our final diagnosis was RRTI caused by underlying PAS. We prescribed RRTI prophylaxis with OM-85 for 3 months and scheduled surgery to correct the PAS as early as possible. Two months later, the patient received the LPA reimplantation and slide tracheoplasty.

The symptoms of respiratory distress resolved following surgery, and the incidence of RTI was reduced. The child experienced an acute mild respiratory infection, likely rhinovirus, in 2014 and mild hand-foot-mouth disease in 2015, for which he received a further 3-month course of OM-85 prophylaxis. In 2017, he received a 3-month prophylactic course of OM-85 without any recurrence of RTI.

## 3. Discussion

To the best of our knowledge, this is the first reported case of PAS presenting with a bronchial foreign body. PAS is a rare cause of respiratory distress with a prevalence of 59/1,000,000 in school-age children [1]. In cases of respiratory distress presenting with a history of RRTI or pneumonia, physicians should consider PAS as a possible diagnosis.

In PAS patients, the anomalous left pulmonary artery originates from the posterior aspect of the right pulmonary artery and passes between the trachea and esophagus forming a sling around the airway before finally reaching the left lung. Because of the nonspecific symptoms, which include respiratory distress manifested by cough, wheezing, cyanosis, and stridor, lack of clinical experience may lead to misdiagnosis and poor outcomes. Use of echocardiogram, followed by CT scan, to rule out PAS should be considered as part of differential diagnosis of infants with respiratory distress [2, 3]. In addition, we should also consider whether there were other anomalies because PAS is often associated with tracheal stenosis and complete tracheal cartilage ring which most commonly causes the symptoms and influences treatment and outcomes.

Pneumonia and RRTI are commonly associated with PAS. We utilized OM-85, which has proven efficacy in reducing RRTI from a Cochrane meta-analysis [4], as prophylaxis in the run up to surgery and in the postsurgical setting. Immunomodulators may be a useful adjunct to control RRTI in these rare cases of congenital PAS.

Surgical correction is essential in children with PAS. To aid preoperative planning and because of the high number of associated congenital abnormalities, preoperative imaging (bronchoscopy, CT with 3D reconstruction, and echocardiogram) is required. In most cases, PAS is best handled via median sternotomy, cardiopulmonary bypass, and left pulmonary artery reimplantation. Cardiac lesions and tracheal stenosis should be repaired simultaneously, where present. In cases where tracheal stenosis is detected, slide tracheoplasty is the preferred option amongst the several techniques available [5, 6].
